# Enhanced Autophagic Flux, Suppressed Apoptosis and Reduced Macrophage Infiltration by Dasatinib in Kidneys of Obese Mice

**DOI:** 10.3390/cells11040746

**Published:** 2022-02-21

**Authors:** Hassan Reda Hassan Elsayed, Randa El-Gamal, Mohammed R. Rabei, Mona G. Elhadidy, Shereen Hamed, Basma H. Othman, Mohamed Mahmoud Abdelraheem Elshaer, Mostafa Khaled Sedky, Ahmed Tarek Abd Elbaset Hassan, Mohammad El-Nablaway

**Affiliations:** 1 Department of Anatomy and Embryology, Faculty of Medicine, Mansoura University, Mansoura 35516, Egypt; 2 Department of Anatomy, Faculty of Medicine, Horus University, New Damietta 34517, Egypt; 3 Department of Medical Biochemistry and Molecular Biology, Faculty of Medicine, Mansoura University, Mansoura 35516, Egypt; drrandaelgamal@mans.edu.eg (R.E.-G.); medo_bio@mans.edu.eg (M.E.-N.); 4 Medical Experimental Research Center (MERC), Faculty of Medicine, Mansoura University, Mansoura 35516, Egypt; Basma_osman_merc22@yahoo.com; 5 Department of Medical Physiology, Faculty of Medicine, Mansoura University, Mansoura 35516, Egypt; ramirabei@mans.edu.eg (M.R.R.); mona.j@bu.edu.sa (M.G.E.); 6 Department of Physiology, Faculty of Medicine, King Salman International University, South Sinai 46511, Egypt; 7 Department of Medical Physiology, College of Medicine, Al-Baha University, Al-Baha 61008, Saudi Arabia; 8 Department of Medical Histology and Cell Biology, Faculty of Medicine, Mansoura University, Mansoura 35516, Egypt; dr_sh_mh@mans.edu.eg; 9 Department of Clinical Pharmacology, Faculty of Medicine, Ain Shams University, Cairo 11566, Egypt; mohamed_elshaer@med.asu.edu.eg; 10 Department of Clinical Pharmacology, Faculty of Medicine, King Salman International University, South Sinai 46511, Egypt; 11 Department of Surgery, Faculty of Medicine, King Salman International University, South Sinai 46511, Egypt; mostafa.sedky@ksiu.edu.eg (M.K.S.); Ahmed.Hassan@ksiu.edu.eg (A.T.A.E.H.); 12 Department of Medical Biochemistry, College of Medicine, Almaarefa University, Riyadh 71666, Saudi Arabia

**Keywords:** macrophages, obesity, Dasatinib, tyrosine kinase inhibitors, autophagy

## Abstract

Obesity causes renal changes (ORC), characterized by defective renal autophagy, lipogenesis, enhanced macrophage infiltration and apoptosis. We hypothesize that Dasatinib, a tyrosine kinase inhibitor, may ameliorate changes associated with obesity. We the mice with either Obesogenic diet (OD) or a standard basal diet. After 12 weeks, the mice received either vehicle or Dasatinib 4 mg/kg/d for an additional four weeks. We examined serum creatinine, urea, lipid profile and renal cortical mRNA expression for lipogenesis marker SREBP1, inflammatory macrophage marker iNOS and fibrosis markers; TGFβ and PDGFA genes; immunohistochemical (IHC) staining for CD68; inflammatory macrophage marker and ASMA; fibrosis marker, LC3 and SQSTM1/P62; autophagy markers and western blotting (WB) for caspase-3; and, as an apoptosis marker, LC3II/I and SQSTM1/P62 in addition to staining for H&E, PAS, Sirius red and histopathological scoring. Dasatinib attenuated renal cortical mRNA expression for SREBP1, iNOS, PDGFA and TGFβ and IHC staining for CD68, ASMA and SQSTM1/P62 and WB for caspase-3 and SQSTM1/P62, while elevating LC3 expression. Moreover, Dasatinib ameliorated ORC; glomerulosclerosis, glomerular expansion, tubular dilatation, vacuolation and casts; inflammatory cellular infiltration; and fibrosis. Dasatinib is a promising therapy for ORC by correcting autophagy impairment, attenuating lipogenesis, apoptosis and macrophage infiltration by inducing antifibrotic activity.

## 1. Introduction

Obesity, independent of the presence of diabetes or hypertension, can contribute to obesity-related renal changes (ORC) characterized by glomerulosclerosis [1] with deformations of several tubular structures of rat kidney and large and congested blood vessels [2]. Moreover, a high fat and carbohydrate diet causes increased renal glycogen accumulation [3] and increased renal inflammatory cells [4]. Furthermore, obesity may cause tubulointerstitial fibrosis; however, the underlying mechanisms remain unknown [5,6,7].

In addition, obesity could induce macrophage polarization towards M1 pro-inflammatory macrophages, rather than M2 anti-inflammatory macrophages, in the adipose tissue, which is proven to play a role in inflammation and insulin resistance, and previous studies have proved that inhibition of pro-inflammatory macrophages succeeds in reducing kidney damage [8]. Dyslipidemia itself causes inflammation, which results in disrupted cell activity and pathological changes in renal tissues [9].

Autophagy is an important process in cell recycling and breakdown of materials for cell homeostasis during different stressful conditions. Altered autophagy in vascular endothelium, epithelium, pericytes, podocytes and mesangium results in imbalance in cell homeostasis. ORC was reported to be associated with suppressed renal autophagy [10].

Dasatinib, a novel highly active oral Bcr-Abl/Src tyrosine kinase inhibitor, was used for the treatment of imatinib-resistant BCR-ABL-positive chronic myelogenous leukemia (CML) as a first line or for patients with resistance to imatinib [11]. Dasatinib could safely treat CML patients with mild to moderate kidney or liver impairment and could reach success rates close to those of CML patients with without organ impairment [12].

Dasatinib was found to suppress steatohepatitis, lipogenesis and serum lipids with the induction of M2 anti-inflammatory macrophage polarization, with inhibition of hepatocyte apoptosis and liver fibrosis in a model of western-diet induced non-alcoholic steatohepatitis [13]. Furthermore, Tyrosine kinase inhibitors (TKIs) were found to induce autophagy in various types of cancer cells in a previous study comparing autophagy-inductive capabilities of many TKIs, by quantitative autophagic flux assay. Among these TKIs, Dasatinib exhibited prominent autophagy induction in A549 and PC-9 cell lines [14].

Most TKIs have relatively nephrotoxic adverse effects. However, CML-Chronic phase patients with mild to moderate renal dysfunction could be safely treated with frontline Dasatinib and achieved response rates similar to those of CML-CP patients with normal organ function [12,15]. Some studies reported adverse renal side effects, especially with high doses, in cancer patients [16]. However, recent studies illustrated the potential of Dasatinib to ameliorate diabetic kidney [17] to delay ischemic renal injury [18] and to inhibit renal fibrosis induced by unilateral ureteral obstruction (UUO) in rats [19].

To the extent of our knowledge, this is the first study to assess the effect of Dasatinib on ORC induced by diet in mice. We proposed that Dasatinib might reverse the histopathological ORC at glomerular and tubular levels, as examined by using Hematoxylin and Eosin (H&E), Periodic acid–Schiff (PAS) and Sirius red-stained kidney sections and that Dasatinib might modulate autophagy at the histopathological and molecular levels. We examined serum creatinine, urea, lipid profile and renal mRNA expression for lipogenesis marker; sterol regulatory element-binding protein 1 (SREBP1), inflammatory macrophage marker; inducible nitric oxide synthase (iNOS), fibrosis markers; transforming growth factor beta (TGFβ) and platelet-derived growth factor subunit A (PDGFA) genes; immunohistochemical (IHC) staining for the macrophage marker CD68; the fibrosis marker; alpha smooth muscle actin (ASMA) with IHC and Western blotting (WB) for the autophagy markers; microtubule-associated proteins light chain 3 (LC3) and sequestosome-1/p62 (SQSTM1/P62); and WB for the apoptosis marker, caspase-3.

## 2. Materials and Methods

### 2.1. Sample Size Estimation

The G*Power program was used to estimate the sample size by using the protocol defined by Faul et al. [20]. According to former studies, our hypothesis is that means, standard deviations and effect sizes (f) and, thus, the sample sizes for the four groups would be considered, as in Table 1, and reach a power of 95% to identify these effect sizes at 5%, alpha level, taking into consideration the least effect size (1.2343) and one-way ANOVA plan with the four groups, suggesting a sample size of four/group. Utilizing an F-test, the total number of 16 for the sample achieves a power of 95% to achieve a significance level of 0.05. 

### 2.2. Experimental Design of the Study, Animals and Diet

**Figure 1:**
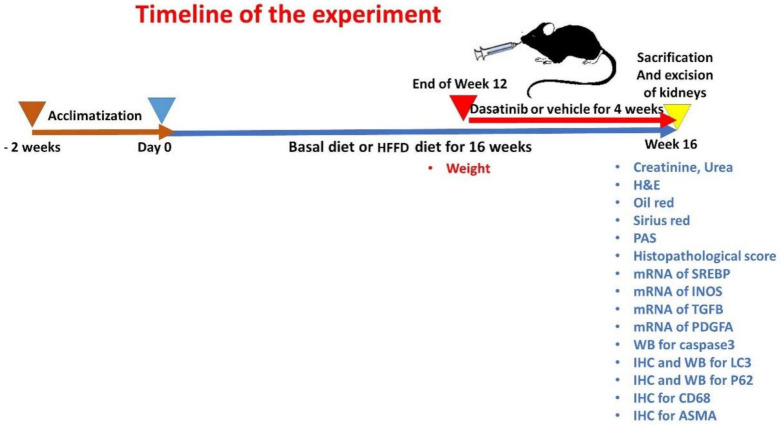
The timeline for the experiment.

Twenty-four adult male C57BL/6 mice of wild type (weighing 20–25 g, 6–7 weeks in age) were obtained from Medical Experimental Research Center (MERC). The experiment was performed following the guidelines of Institutional Research Board (IRB), Faculty of Medicine, Mansoura University, Egypt (proposal code: R.20.12.1104.R1). Mice were applied because they have mammalian physiological systems mimicking those of humans, and males are more vulnerable to diet-induced obesity [27]. All animals were housed in MERC in stainless-steel cages under precise pathogen-free conditions (four mice/cage). All animals were kept in an environmentally controlled facility having a natural 12/12 light/dark cycle, temperature and humidity. The animals were housed for two weeks prior to the start of experiment to allow acclimatization. The mice were divided in a randomized manner into two main groups: one fed a basal Diet (BD) (n = 12) and the other one was fed an obesogenic diet (OD) (Calories; 45.3% from fat, 37% from Carbohydrates and 17.7% from protein) to induce ORC (n = 12) in accordance with the detailed diet composition used by Elsayed et al. [13]. The weights of animals were recorded at the end of the 12th week to confirm establishment of weight gain and obesity. Mice with weight >3 standard deviations of the control group were considered obese [28]. After 12 weeks, the BD (n = 12) group was randomized to 2 groups: Group 1 (n = 6) received dimethyl sulfoxide (DMSO) 5% in PBS, and Group 2 (n = 6) received Dasatinib 4 mg/kg through 4 weeks. Moreover, the OD group (n = 12) was randomized into 2 groups: Group 3 (n = 6) received 5% DMSO in PBS, and Group 4 (n = 6) received Dasatinib 4 mg/kg body weight for additional four weeks. The dose of Dasatinib was selected based on the earlier work of Hassan et al. [19], who reported that Dasatinib at a daily dose of 4 mg/kg slowed the progression of renal interstitial fibrosis via attenuating renal oxidative stress. The mice were then weighed and anaesthetized by ether inhalation then sacrificed by cervical dislocation. Both Dasatinib and DMSO were administered through oral gavage. The timeline for the study is demonstrated in Figure 1.

### 2.3. Drug Preparation

Dasatinib (Sigma Aldrich C.N. SML2589, Steinheim, Germany) was melted in 5% DMSO, as a stock solution, and then it was distributed into aliquots. Until being used, it was stored in −20 °C. Right before use, the dilution of Dasatinib was conducted in PBS to a concentration of 2 mg/mL. Dasatinib measuring 4 mg/kg was administrated to the Dasatinib control group and OD + Dasatinib group by oral gavage once daily. The Dasatinib dose was determined in accordance to previous studies [19].

### 2.4. Blood and Tissue Sampling

Mice were subjected to 12 h overnight fasting (18:00 p.m.–6:00 a.m.) at the last night of the experiment. Mice were subjected to ether inhalation then sacrificed through cervical dislocation. Kidneys were rapidly excised, washed and then dried and weighed. Samples of renal cortices were used because the renal cortex is more affected by obesity [1]. The blood samples were obtained from hearts of mice and placed in EDTA-free tubes. Sera were separated and stored at −20 °C until analysis is performed. 

### 2.5. Assessment of Glucose, Kidney Function Tests and Lipid Profile

At the end of experiment, mice sera were assessed for fasting glucose level, kidney function tests and lipid profile by colorimetric assay using glucose, urea, creatinine, triglycerides, total cholesterol and high density lipoprotein cholesterol (HDL-C) endpoint kits (MG, Cairo, Egypt) and also low density lipoprotein (LDL) cholesterol was calculated following the procedures and calculation described in the previous study of Elsayed et al. [13].

### 2.6. mRNA Quantification of the Studied Genes

Overnight at 2–8 °C, samples of the renal tissue were kept in RNA later (Qiagen, Erlangen, Germany) and then stored in −80 °C until being processed. Renal tissue samples were subjected to homogenization by liquid nitrogen. RNA was extracted utilizing QIAzol reagent (Qiagen, Erlangen, Germany). RNA product was tested by Thermo Scientific NanoDrop 2000 (Thermofischer Scientific, Wilmington, NC, USA) [29]. Utilizing SensiFAST™ cDNA Synthesis Kit (Bioline, London, UK), reverse transcription of 1 ug of RNA was performed (10 min at 25 °C for primer annealing, 15 min at 42 °C for reverse transcription and 85 °C for 5 min for inactivation) on an Applied Biosystems 2720 Thermal Cycler. 

Quantitive real time PCR (qRT-PCR) was carried out using SYBR green master mix (Willowofort, Birmingham, UK) in a total reaction volume of 20 µL utilizing a real-time PCR instrument (Pikoreal 96, ThermoScientific, Wilmington, NC, USA: 95 °C for 2 min, 40 cycles of 95 °C for 10s and 60 °C for 30 s [30]. The sequences of the used primer pairs were supplied in our previous study by Elsayed et al. [13]. Glyceraldehyde-3-phosphate dehydrogenase (GAPDH) was applied as a control gene. The primer sets were examined for specificity utilizing Primer-BLAST program [31]. The specificity of the PCR products was also assessed by melting curve analysis. Primers sets were produced by Vivantis (Vivantis Technologies, Selangor, Malaysia). Relative gene expression levels were presented as ΔCt = Ct target gene–Ct housekeeping gene; the fold change of gene expression was estimated following the 2^−ΔΔCT^ method [32].

### 2.7. Detection of the Studied Proteins Expression by Western Blotting

Using QIAzol reagent, the extraction of total protein from renal cortical samples was performed according to the company’s specifications. After that, protein concentrations were quantified using Bradford protein assay (Bosterbio, Pleasanton, CA, USA). Of each sample, 20 µg equal amounts was separated together with a pre-stained protein molecular weight marker (Bio-Rad, Hercules, CA, USA) via 10% SDS-PAGE [33]. After separation, protein transfer to 0.22 µm nitrocellulose membrane (Abcam, Boston, MA, USA) was performed by using Eco-Line Biometra apparatus (Gottingen, Germany); then, the membranes were incubated in 5% non-fat milk blocking buffer for 1 h at 37 °C. After blocking, an overnight membrane incubation with the primary antibodies—rabbit polyclonal antibody for LC3 and rabbit polyclonal antibody for SQSTM1/P62 as markers for autophagic activity, mouse monoclonal antibody for caspase-3 as an apoptosis marker and β-actin as a control protein (Biospes; YPA1652, ABclonal; A11250, Santa Cruz sc-7272 and Abcam ab227387, respectively)—was performed overnight at 4 °C (1:500, 1:1000, 1:100 and 1:5000 dilutions, respectively). Dilution of primary antibodies was performed in a blocking buffer, followed by incubation in an HRP-conjugated secondary anti-mouse IgG or anti-rabbit IgG antibodies (sc-516102 or sc-2357, Santa Cruz, respectively) for 1 h at room temperature [34]. The chemiluminescent substrate (ClarityTM Western ECL substrate Bio-Rad, Hercules, CA, USA) was added to the blot following the guidelines of the manufacturer, and then the chemiluminescent signal was photographed using a CCD camera-based imager. Assessment of target proteins band intensity against control protein β-actin was performed using ChemiDoc MP imager.

### 2.8. Histopathology

A part of the renal cortex of each kidney was subjected to fixation in 10% formaldehyde then kept in paraffin [35] for histopathological investigation. After that, the paraffin blocks were dissected into 5–7-micrometer-thick sections. The sections were subjected to staining with (H&E) for assessment of histopathological alteration and by Sirius red to identify the degree of the fibrosis and PAS stain for glycogen. Photomicrography was performed using Olympus Microscope with SC100 camera (Shinjuku, Tokyo, Japan).

### 2.9. Histopathological Assessment of Renal Cortical Damage

The histopathological semiquantitative score for renal cortical damage was performed to estimate the degree of damage. The score was quantified in 24 simple random non-overlapping microscopic fields, scattered in the preparations as a representative sample (2 fields per each section of 2 sections per mouse for each mouse of the 6 mice per group) from H&E, Sirius red and PAS-stained kidney sections of the mice. We assessed six characters: the degree of glomerulosclerosis, tubular vacuolation, tubular dilatation, cast formation, the number of inflammatory cells and extent of fibrosis [36]. A score of 0 was considered when the section shows no damage; a score of 1 was considered when less than 20% was present; a score of 2 was considered when there was at least 20% but less than 35%; a score of 3 was considered when there was at least 35% but less than 50%; a score of 4 was considered when there was at least 50% but less than 65%; a score of 5 was considered when there was at least 65% but less than 80%; and, finally, a score of 6 was considered when there was at least 80%.

### 2.10. Immunohistochemical Staining

Immunohistochemistry was performed on 3-micrometer-thick paraffin sections following the immunoperoxidase technique applied by Elsayed et al. [13]. In brief, the sections were deparaffinized and hydrogen peroxide with (0.3%/methanol) was added to stop endogenous peroxidase activities at room temperature for 10 min. Then, the sections were heated for 10 min at 95 °C in 10 mM citrate buffer to induce antigen retrieval, and then they were left to cool for 1 h. Kidney sections were kept with the primary rabbit monoclonal antibody for CD68 as a marker for macrophage, rabbit polyclonal antibody for LC3 and rabbit polyclonal antibody for SQSTM1/P62 as markers for autophagic activity and mouse monoclonal antibody for ASMA as a fibrogenic marker (Genemed; 60-0184, Biospes; YPA1652, ABclonal; A11250 and Biolegend MMS-466S, respectively) overnight at 4 °C (1:100, 1:400, 1:200 and 1:100 dilutions, respectively). The slides were incubated with universal mouse/rabbit polydetector plus (BSB 0257, Bio SB) for 30 min. After that, DAB was added for 4 min, and then the slides were counterstained by hematoxylin. For reagent control, PBS was added to replace the primary antibodies. Finally, the sections were then subjected to washing, dehydration and examination by light microscope [37]. Dark brown cytoplasmic areas demonstrate positive staining for CD68, LC3, P62 and ASMA while the background is blue.

### 2.11. Morphometric Analysis of Immunohistochemical Results

The percentage of the LC3, P62 and ASMA immunopositive area fractions as and the number of CD68 immunopositive cells per high power field were quantified (in ×400) using ImageJ software version (1.52a) [38] and Fiji Imagej software [39]. In brief, to measure immunopositive area fractions, the color deconvolution plugin tool was used and H-DAB vector was selected and resulted in three images. In the brown channel, we set the threshold at 0–85 (Image → Adjust → Threshold). We recorded the area fraction (Analyze → Measure → Area fraction). Quantification was pwerformed for 24 simple non-overlapping random microscopic fields, scattered in the preparations as a representative sample (2 fields per each section of 2 sections per mouse for each mouse of the 6 mice per group). 

### 2.12. Statistical Analysis

The results were evaluated using IBM-SPSS software (Version 25.0., IBM Corp, Armonk, NY, USA). In the beginning, quantitative data were verified for normality utilizing Shapiro–Wilk’s test. Quantitative data were presented as mean ± standard error (SE) when normally distributed. One-Way ANOVA test with LSD post hoc analysis were applied to compare among quantitative normally distributed data. Non-normal data were demonstrated as median and interquartile range and the Kruskal–Wallis H test was used to compare them. The results were considered significant when *p* value ≤ 0.050. 

## 3. Results

### 3.1. Results of Final Body Weights and Kidney Weights

A study of final body weights and kidney weights showed statistically significant differences between the four study groups (*p*: <0.013 and 0.001, respectively). They showed significant elevated values in OD group versus other groups. They showed significant elevation in the OD group when compared to the control groups. Kidney weights were significantly lowered in the Dasatinib-treated group when compared to the OD group and a non-significant difference from the control groups was observed, while body weights of dasatinib treated group were insignificantly lowered as compared to the OD group, with insignificant differences from the control group (Table 2).

### 3.2. Results of Fasting Glucose Level and Renal Function Tests

The study of biochemical parameters in serum including glucose, urea and creatinine showed statistically significant differences between the four study groups (*p*: <0.0005, <0.001 and <0.0005, respectively). There was a significant higher level of serum urea, and creatinine in OD group versus other groups. Regarding glucose, urea and creatinine, they showed a statistically significant increased level in the OD group as compared to the control groups, but their levels were lowered in the Dasatinib-treated group; however, glucose and urea levels were still showing a statistically significant higher level than that in the control group, while creatinine showed no significant difference from control groups Table 3.

### 3.3. Effect of Dasatinib on Lipid Profile in Obese Mice

Lipid profile was significantly increased in the OD group as compared to other groups (*p* < 0.0005) Table 4. This result was reversed in the group treated with Dasatinib, with a significant decrease in all parameters of lipid profile when compared with the OD group except for HDL-C levels that showed a non-significant reduction in the Dasatinib-treated group.

### 3.4. The Expression Profile of iNOS, TGF-β, SREBP and PDGFA

**Figure 2:**
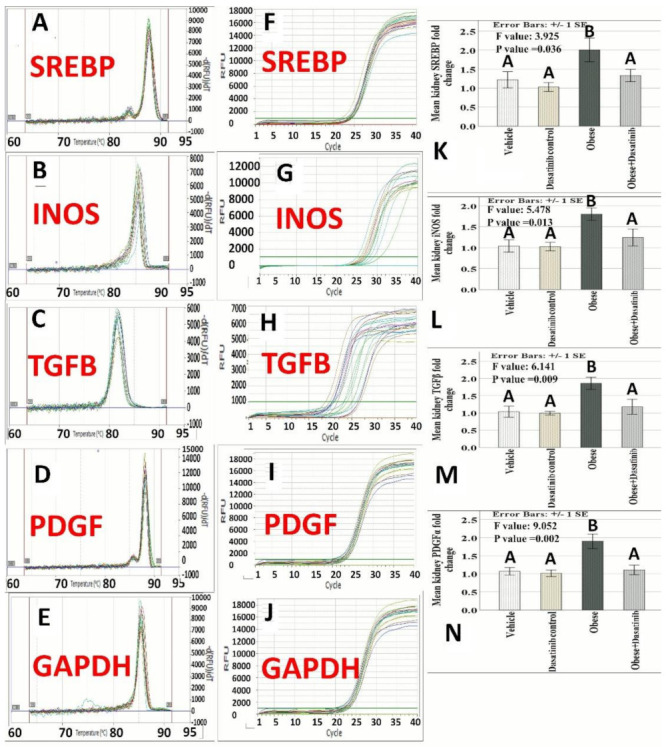
Effect of Dasatinib on melting curves, amplification plotsand histograms of gene expression profiles of SREBP, iNOS, TGF-β, PDGFα and GAPDH genes as studied by qRT-PCR in the model of obeosogenic diet-induced renal damage in mice. (**A**–**E**): Melting curves of the studied genes showing single sharp peaks confirming primers specificity. (**F**–**J**): Amplification plots “Linear view” showing the threshold which was set above the baseline in the exponential portion of the plot to accurately determine the threshold cycle (C_t_) of the studied genes. (**K**–**N**): Histograms of the studied genes expression showing the means ± standard errors (SE) of genes fold change. Data are mentioned as mean ± SE, different letters = significant difference. SREBP1: Sterol regulatory element binding protein; iNOS: Inducible nitric oxide synthase; TGF-β: Transforming growth factor beta; PDGFA: Platelet-derived growth factor alpha.

There was a statistically significant difference in iNOS, TGF-β, SREBP and PDGFA levels among the four groups (*p* = 0.013, 0.009, 0.036 and 0.002, respectively). Genetic profiles in OD group showed higher expression as compared to other groups. Although levels in the OD + Dasatinib group were higher than negative control group, these differences were not statistically significant. The amplification plots, melting curves and histograms of gene expression profiles as studied by qRT-PCR are shown in Figure 2.

### 3.5. Effect of Dasatinib on Protein Expression as Assessed by Western Blotting

**Figure 3:**
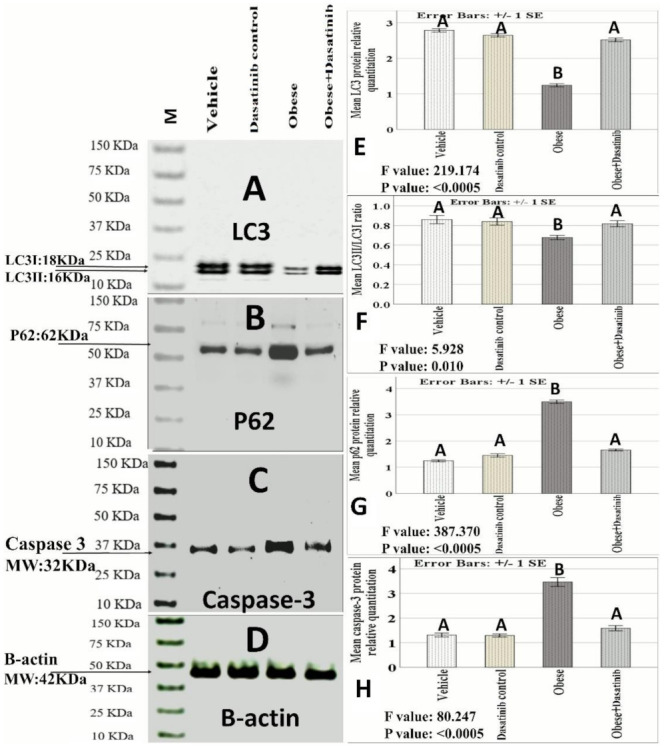
(**A**–**D**) Protein analysis by Western blotting. (**A**) LC3 I and II (Molecular weight: 18 kDa and 16 kDa, respectively). (**B**) p62 (Molecular weight: 62 kDa). (**C**) Caspase-3: 32 kDa). (**D**) β-actin: 42 kDa). (**E**–**H**): LC3II, LC3II/LC3I ratio, P62 and caspase3 proteins relative quantitation /β-actin ratio) by Western blotting. Data are mentioned as mean ± SE; different letters = significant difference). LC3: Microtubule-associated proteins 1A/1B light chain 3B. M: Marker. MW: molecular weight.

It revealed a highly significant difference in LC3, P62 and caspase3 expressions between the four groups (*p* < 0.0005). Pairwise comparisons showed a significant increase in caspase-3 and p62 in the OD-fed group but a significant reduction in LC3II versus other groups. There was a significant decrease in caspase-3 and p62 protein levels with significant increase in LC3II in the OD + Dasatinib group as compared to the OD-fed group. Regarding caspase-3, it showed a significant decrease in the Dasatinib-treated OD groups than the untreated OD group but showed no significant difference between the Dasatinib-treated group and control groups. As for LC3II, it showed a significant elevation in the Dasatinib-treated OD group as compared to the untreated OD group, with no significant difference between that group and Dasatinib control group. Regarding the ratio between LC3II/LCI protein expression, it was significantly lower in the untreated OD group than the control groups, with no significant differences between the Dasatinib-treated group and the control groups. Regarding p62 protein expression, it showed a significant decrease in the treated OD groups as compared to the untreated OD group, with a significant difference between that group and the control groups (Figure 3).

### 3.6. Effect of Dasatinib on Renal Histopathological Changes Induced by OD

**Figure 4:**
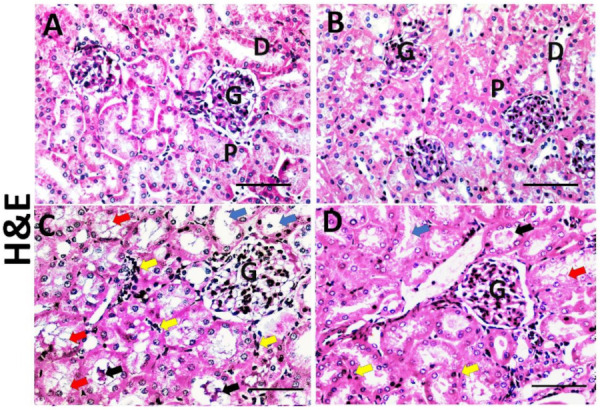
Representative photographs of kidney histopathology; haematoxylin and eosin (×400) (**A**–**D**) in kidney tissues of vehicle, Dasatinib control, Obesogenic diet and Obesogenic diet + Dasatinib groups (**A**–**D**, respectively). ×400. Scale bar = 50 µm. Vehicle group and Dasatinib control group show normal kidney cortical architecture (**A**,**B**), normal glomeruli (G), proximal convoluted tubules (P) and distal convoluted tubules (D), while the Obesogenic diet group (**C**) showed distortion of kidney architecture, glomerular expansion (G), tubular vacuolation (red arrows), tubular dilation (blue arrows), cast formation (black arrows) and increased number of inflammatory cells (yellow arrows). However, Obesogenic diet + Dasatinib group (D) showed relative restoration of kidney architecture with mild glomerular expansion (G), less marked tubular vacuolation (red arrows), occasional tubular dilation (blue arrows), few cast formation (black arrows) and few number of inflammatory cells (yellow arrows).

**Figure 5:**
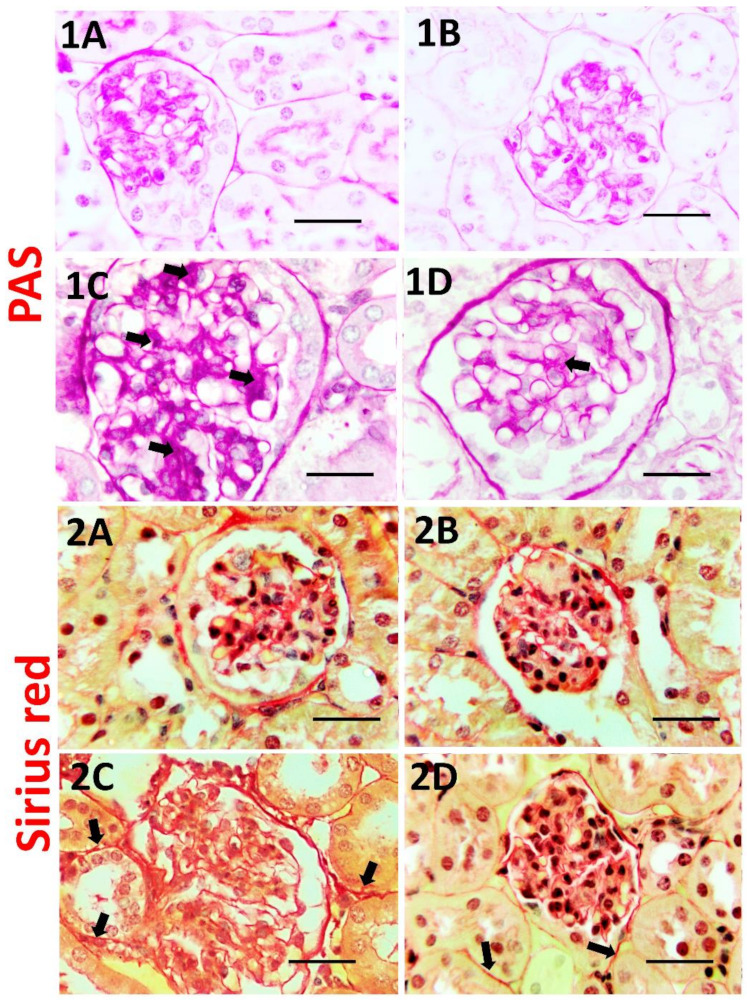
PAS staining for glycogen and mucopolysaccharides (**1A**–**1D**) in kidney tissues of vehicle, Dasatinib control, Obesogenic diet and Obesogenic diet + Dasatinib groups (**1A**–**1D**, respectively). Magnification (×1000). Scale bar = 20 µm. It showed a mild staining for glycogen and mucopolysacharides in the glomeruli (arrows) in vehicle and Dasatinib control groups (**1A**,**B**), strong reaction with glomerulosclerosis (arrows) in Obesogenic diet group (**1C**) and moderate reaction in obesogenic diet+ Dasatinib group (**1D**). Sirius red staining for collagen (**2A**–**2D**) in kidney tissues of vehicle, Dasatinib control, Obesogenic diet and Obesogenic diet + Dasatinib groups (**1A**–**1D**, respectively) (×1000) magnification. Scale bar = 20 µm. It shows mild reaction for collagen in vehicle and Dasatinib control groups (**2A**,**B**), strong reaction in Obesogenic diet group (**2C**) and moderate reaction in obesogenic diet+ Dasatinib group (**2D**). Arrows = fibrosis.

H&E of the vehicle and Dasatinib controls showed normal kidney cortical architecture, while the Obesogenic diet group showed distortion of kidney architecture, glomerular expansion, tubular dilation, tubular vacuolation, cast formation and increased number of inflammatory cells. However, the Obesogenic diet + Dasatinib group showed relative restoration of kidney structure Figure 4. Sirius red showed mild staining of collagen between the tubules in vehicle and Dasatinib control groups and strong staining in the OD group with moderate staining in OD + Dasatinib group Figure 5. PAS staining showed normal staining in control groups, strong staining with glomerulosclerosis in the OD group and moderate staining in the Dasatinib-treated obese mice group (Figure 5).

### 3.7. The Histopathological Score

The histopathological score among the groups showed a significant difference with higher scores in the OD group (median value = 19.5) when compared to vehicle and Dasatinib control groups (median value = 3), with significant reduction in OD + Dasatinib group (median value = 9) when compared to the OD group (Table 5).

### 3.8. Immunohistochemical Results

**Figure 6:**
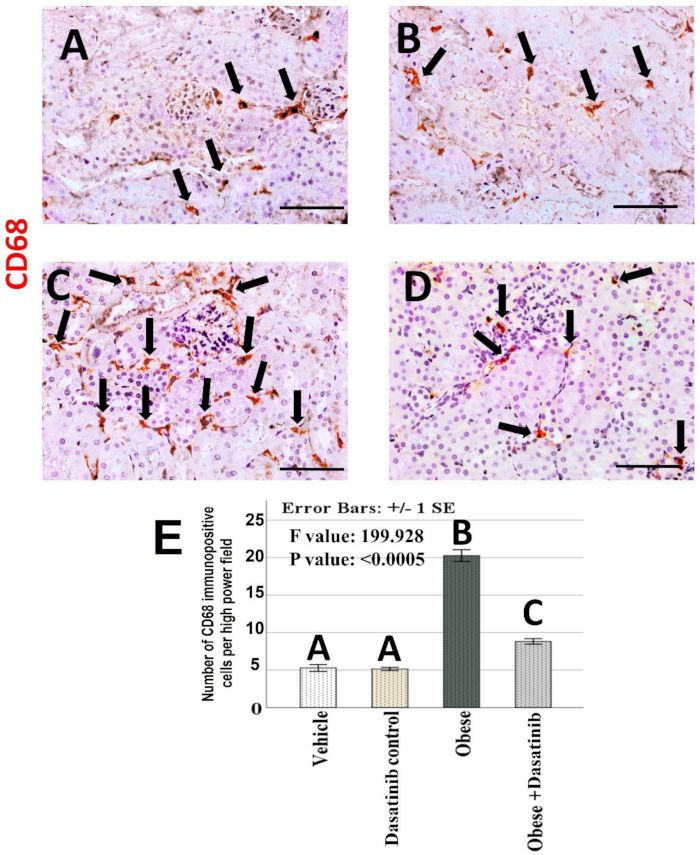
(**A**–**D**): Immunohistochemical staining for CD68 (×400) in kidney tissues of vehicle, Dasatinib control, Obesogenic diet and Obesogenic diet + Dasatinib groups (**A**–**D**, respectively). Scale bar = 50 µm. (**E**) The number of CD68 immunopositive cells per high power fields. Results are mentioned as mean ± standard error. Different letters mean significant difference. Arrows = CD68 + ve cells.

**Figure 7:**
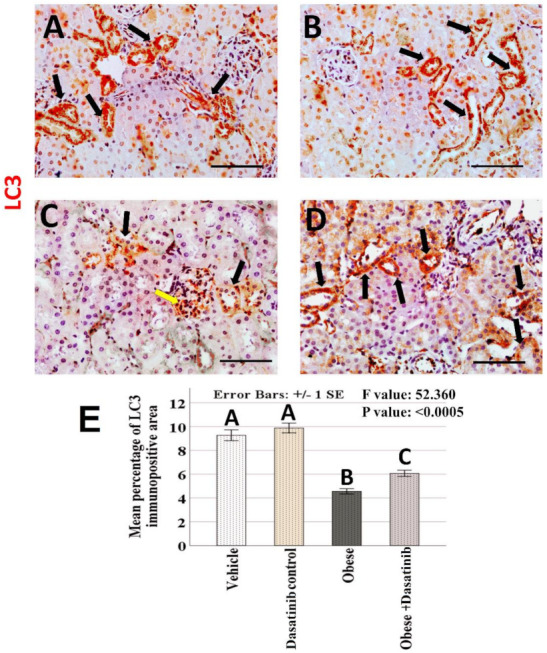
(**A**–**D**): Immunohistochemical staining for LC3 (×400) in the kidney tissues of vehicle, Dasatinib control, Obesogenic diet and Obesogenic diet + Dasatinib groups (**A**–**D**, respectively). Scale bar = 50 µm. (**E**) The mean percentage of LC3 immunopositive area. Results are presented as mean ± standard error. Different letters mean significant difference. Arrows: LC3 + ve cells. LC3 = Microtubule-associated protein light chain 3.

**Figure 8:**
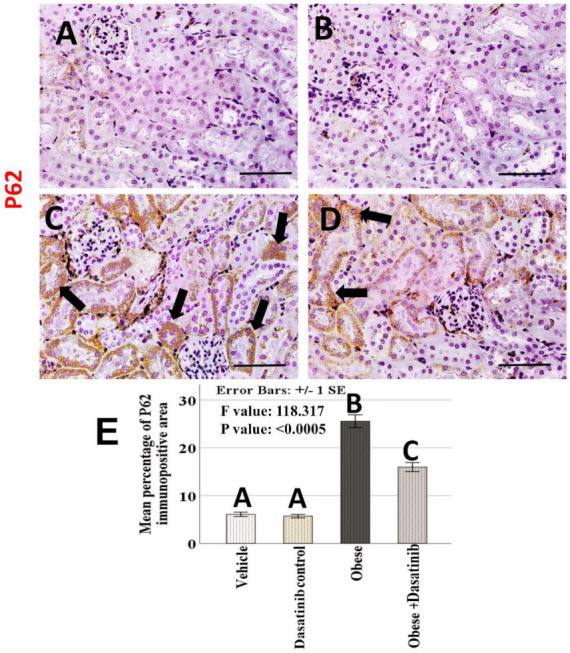
(**A**–**D**): Light-microscopic image of renal tissue with P62 immunopositive staining (×400) in the kidney tissues of all experimental groups; vehicle, Dasatinib control, Obesogenic diet and Obesogenic diet + Dasatinib groups (**A**–**D**, respectively). Scale bar = 50 µm. (**E**) Morphometric analysis of the mean percentage of P62 immunopositive area. Results are mentioned as mean ± standard error. *p* value is presented as letters (different letters mean statistically significant difference). Arrows = P62 + ve cells.

**Figure 9:**
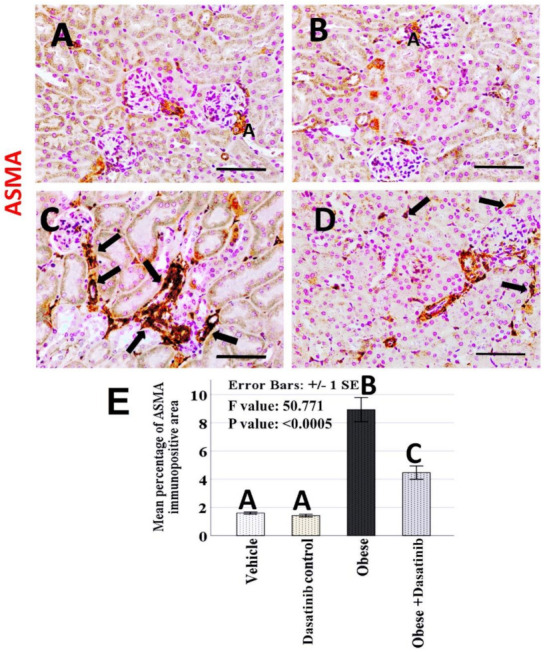
(**A**–**D**): Immunohistochemical staining for ASMA (×400) in the kidney tissues of vehicle, Dasatinib control, Obesogenic diet and Obesogenic diet + Dasatinib groups (**A**–**D**, respectively). Scale bar = 50 µm. (**E**) The mean percentage of ASMA immunopositive area. Results are presented as mean ± standard error. Different letters mean statistically significant difference. Arrows: ASMA + ve cells. ASMA = Alpha smooth muscle actin.

Weak CD68+ve expression was observed in control groups. Kidneys of OD group showed an intense expression for CD68 in the interstitial cells between tubules as a marker for macrophages. In contrast, immunoreactivity was reversed in the Dasatinib-treated group, showing a weak expression in Figure 6. However, LC3 immunoreactivity showed an inverted manner for expression than compared to CD68; it showed moderate staining in the renal tubules in control groups, mild staining in OD group and moderate staining in Dasatinib-treated obese mice group Figure 7. Furthermore, weak P62+ve expression was observed in control groups. Kidneys of OD group showed an intense expression for P62 in the renal tubular cells. In contrast, immunoreactivity was reversed in Dasatinib-treated group, showing a weak expression Figure 8. Lastly, mild ASMA+ve expression was observed in the walls of renal arterioles in kidney sections of control groups. Noticeable, kidneys of the OD group showed additional intense expression in the renal interstitium between tubules. In contrast, immunoreactivity was reversed in Dasatinib-treated group showing a weak expression for ASMA in Figure 9.

### 3.9. Morphometric Assessment of Immunohistochemical Findings

The mean and standard error of percentage of LC3, P62 and ASMA immunopositive areas and the number of CD68 immunopositive cells showed significant difference among groups (*p* < 0.0005) (Figure 6, Figure 7, Figure 8 and Figure 9). LSD post hoc analysis for the mentioned proteins showed no significant difference between vehicle and Dasatinib control groups and also showed that the percentage of the immunopositive area was significantly higher in OD group when compared to the other groups, with a significant decrease in OD + Dasatinib group except for LC3, which showed the opposite; it showed the most significant reduction in OD group, which was increased significantly in Dasatinib-treated obese mice group.

## 4. Discussion

In the current study, the induction of ORC was performed by obesogenic diet (OD) in mice as confirmed by H&E, PAS, Sirius red-stained kidney sections. Similar to the results of previous studies, the OD, caused renal steatosis [40], tubular damage and lipid droplets accumulation in tubular cells [41], increased renal fibrosis and collagen accumulation and glycogen accumulation [3] and increased number of inflammatory cells may be associated with fat accumulation in the kidney [4]. Moreover, kidney disease in obesity has been attributed to the impact that visceral adiposity results in physical compression on the kidney [42].

Moreover, OD caused an increase in serum creatinine and urea [43], and serum lipids [40] and this dyslipidemia itself stimulate inflammation, which results in disrupted cell activities and pathological changes in renal tissues [9]. Obesogenic diet caused distortion of kidney architecture, glomerulosclerosis, tubular dilation and vacuolation, cast formation, renal steatosis, increased number of inflammatory cells and tubulointerstitial fibrosis. OD also increased renal lipogenesis marker SREBP1 [40] and renal inflammatory macrophage proinflammatory markers iNOS and CD68 in kidney tissues [3]. Furthermore, defective autophagy was confirmed by decreased LC3 and increased P62 expressions [10]. Moreover, it was associated with an elevation in renal fibrosis markers: TGF-β, PDGFA [5] and ASMA [6]. Tyrosine kinases have been found to play an important role in the inflammatory signaling pathways, triggered by free radicals associated with obesogenic diet [44,45].

To the best of our knowledge, this may be the first study to assess the role of Dasatinib, a tyrosine kinase inhibitor, on ORC. The Dasatinib dose was determined in accordance of previous studies [19] and Elsayed et al. [13]. Successfully, Dasatinib could reverse ORC, renal fat infiltration, inflammatory and fibrotic changes produced by OD, and this confirms the ability of Dasatinib to preserve kidney tissues and attenuate steatosis, inflammation, fibrosis and modulate autophagy at the histopathological level.

In the current study, Dasatinib could attenuate the OD-induced elevation of serum total cholesterol, LDL and TG, similar to the findings of Elsayed et al. [13]. Furthermore, we found a decrease in SREBP-1 in the OD + Dasatinib group as Dasatinib functions as a PDGFR inhibitor, as it is suggested that PDGF stimulates membrane lipid production via triggering of SREBP [46], a result that can be stopped by Dasatinib.

In our experiment, Dasatinib could correct autophagic machinery as observed with the increasing expression of LC3II and LC3II/LC3I ratio and decreasing expression of P62 by inhibiting apoptosis, as observed by decreased caspase-3 expression; these findings coincide with the findings of Tanaka et al. [14], who compared the autophagy-inductive capabilities of many TKIs by quantitative autophagic flux assay. Among these TKIs, Dasatinib exhibited prominent autophagy induction in A549 and PC-9 cell lines. Moreover, Elsayed et al. [47] found that the induction of autophagy could attenuate high fat and fructose diet-induced hepatic injury. Furthermore, Sohn et al. [48] stated that stimulation of autophagy by fatty diet rescued the kidney from injury. In addition, deletions of autophagy-related genes have been found to trigger proteinuria and podocyte injury [49,50], indicating that autophagy is involved in maintenance of normal renal function. 

In the present study, Dasatinib could suppress the number of inflammatory macrophages as observed by the decreased expression of renal iNOS and CD68 in kidney, and this may be similar to the findings of Cruz et al. [51], who reported similar effects resulting in improvement of lung functions in an experimental model of silicosis and, thus, reducing inflammation and pulmonary fibrosis. Moreover, Elsayed et al. [13] found that Dasatinib could induce macrophage polarization towards decreasing inflammatory M1 macrophage and increasing M2 macrophage in a model of non-alcoholic steatohepatitis, as observed through downregulation of CD68 and iNOS with upregulation of CD163 and Arginase 1, respectively, thus deceasing hepatic inflammation and fibrosis. 

Our finding of the Dasatinib effect in decreasing renal fibrosis as observed through Sirius red staining and through a reduction in fibrogenic mediators, PDGFA, TGFβ and also decreased ASMA, is in accordance with the results of Hassan et al. [19], who reported slow progression of renal interstitial fibrosis induced by unilateral ureteral obstruction (UUO) in rats, possibly via suppressing renal oxidative stress, impairing Src/STAT-3/NF-kappaB signaling, reducing renal inflammation and, thus, reducing fibrogenic mediators: PDGFA, TGFβ and ASMA. Moreover, Dasatinib considerably suppressed PDGFA and TGF-β-induced phosphorylation of ERK and Akt in previous studies [13,52].

Dasatinib and Quercetin, which constitute senolytic therapy, could suppress renal senescence and attenuate renal fibrosis in both unilateral renal ischemia/reperfusion and multiple-cisplatin treatment models; thus, they might be promising for treating chronic kidney disease after acute kidney injury [53]. Furthermore, senolytic therapy could modulate glucose tolerance and lipid metabolism, with the suppression of adipose tissue inflammation, T lymphocyte cellular infiltration, reduction in senescent cells and attenuation of hepatic gluconeogenesis in old mice [54]. Moreover, Src inhibition by Dasatinib was found to increase the accumulation of apical membrane Aquaporin 2 (AQP2) in the principal cells of the collecting duct, thus regulating water reabsorption, and this might be a different mechanism underlying the therapeutic effect of Dasatinib in ORC [55]. 

Questions were raised concerning the cardiovascular side effects of Dasatinib. However, these complications and alterations are potentially rare, especially with prolonged use, daily treatment and high doses as in the treatment of chronic myelogenous leukemia. They include prolongation of the QT interval, arrhythmia and palpitations [56]. On the other hand, low-dose Dasatinib was found to ameliorate hypertrophic cardiomyopathy progression [57] so that the use of Dasatinib in low dose and short time is relatively safe. C57BL/6 male mice were used because they have mammalian physiological systems mimicking those of humans, and males are more vulnerable to diet-induced obesity [27]. However, some of the reasons for the inconsistent results of obesogenic diet on kidney may be the animal or species difference. In addition, the effect may vary with the prolongation of the duration of model and the duration of the treatment. Furthermore. The sex difference due to the effect of different sex hormones affecting metabolism and endocrine system may also cause controversial results. Thus, it was suggested to repeat the study with different durations of treatment, with different animals and species in different sexes.

## 5. Conclusions

**Figure 10:**
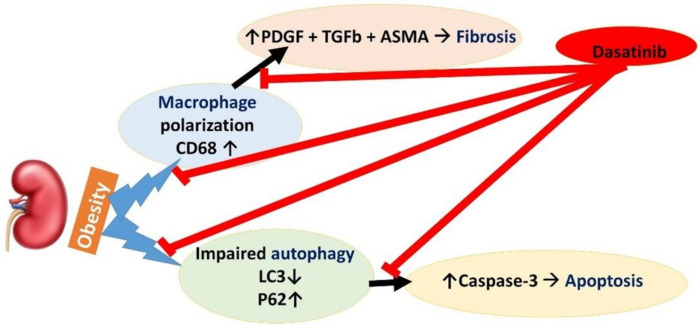
A graphical scheme for the findings of the study.

To date, there is no obviously efficient treatment for obesity-related renal changes (ORC). In this study, Dasatinib has been validated to correct autophagy impairment and attenuate lipogenesis, apoptosis and macrophage infiltration with inducing antifibrotic activity. Dasatinib is suggested to be a remarkable option for the management of ORC. Figure 10 represents a graphical scheme for the study’s findings.

## Figures and Tables

**Table 1:** Sample size calculation.

Studied Parameter	Reference	Means	Standard Deviations	Effect Sizes	Sample Sizes
CD68	[21]	2, 11, 11 and 27	2	4.5052	8
iNOS	[22]	1.1, 2.2, 2.2, and 5.2	0.6	2.5424	8
LC3	[23]	0.8, 1.3, 1.3, and 1.4	0.19	1.2343	16
P62	[23]	0.22, 0.25, 0.25 and 0.31	0.025	1.3077	16
SREBP	[24]	0.4, 1, 1, and 1.2	0.08	3.75	8
TGF-b	[25]	1, 1.1, 1.1 and 1.5	0.15	1.2802	16
ASMA	[26]	8, 11, 17 and 26	2	3.4369	8

**Table 2:** Final Body weights and kidney weights.

Parameter	Group				F Value	*p* Value
	Vehicle (n = 6)	Dasatinib Control (n = 6)	Obese (n = 6)	Obese + Dasatinib (n = 6)		
Body weight (gm)	34 ± 1.83 A	33 ± 1.63 A	41.25 ± 1.11 B	37.25 ± 1.65 AB	5.565	0.013
Kidney weight (gm)	0.24 ± 0.012 A	0.24 ± 0.014 A	0.32 ± 0.009 B	0.27 ± 0.009 A	10.114	0.001

Data are tabulated as mean ± standard error. Different letters = statistically significant difference. significant *p* values are considered if ≤0.05.

**Table 3:** Serum biochemical parameters.

Parameter	Group				F Value	*p* Value
	Vehicle (n = 6)	Dasatinib Control (n = 6)	Obese (n = 6)	Obese + Dasatinib (n = 6)		
Glucose (mg/dL)	83.25 ± 3.12 A	85.50 ± 1.71 A	121.50 ± 1.55 B	96.00 ± 2.74 C	44.780	<0.0005
Creatinine (mg/dL)	0.088 ± 0.012 A	0.082 ± 0.015 A	0.188 ± 0.018 B	0.098 ± 0.009 A	13.058	<0.0005
Urea (mg/dL)	32.98 ± 4.12 A	38.05 ± 4.52 A	72.54 ± 6.02 B	56.10 ± 5.90 C	12.032	0.001

Data are tabulated as mean ± standard error. Different letters = statistically significant difference. Significant *p* values are considered if ≤0.05.

**Table 4:** Serum lipid profile.

Parameter	Group				F Value	*p* Value
	Vehicle (n = 6)	Dasatinib Control (n = 6)	Obese (n = 6)	Obese + Dasatinib (n = 6)		
Total cholesterol (mg/dL)	70.88 ± 6.99 A	72.62 ± 3.15 A	201.75 ± 8.19 B	124.64 ± 4.66 C	102.481	<0.0005
Triglyceride (mg/dL)	90.34 ± 4.17 A	93.92 ± 6.12 A	167.23 ± 6.72 B	130.22 ± 1.56 C	50.705	<0.0005
LDL (mg/dL)	32.76 ± 5.83 A	34.58 ± 5.04 A	127.53 ± 6.95 B	65.27 ± 3.58 C	65.032	<0.0005
HDL-C (mg/dL)	27.45 ± 1.39 A	27.98 ± 1.46 A	42.98 ± 2.88 B	39.82 ± 1.80 B	16.491	<0.0005

Data are tabulated as mean ± standard error. Different letters = significant difference.

**Table 5:** Results of histopathological score of kidney injury.

Parameter	Group				F Value	*p* Value
	Vehicle (n = 6)	Dasatinib Control (n = 6)	Obese (n = 6)	Obese + Dasatinib (n = 6)		
Glomerulosclerosis	0(0–1) A	0(0–1) A	4(2–4) B	2(1–2) C	50.523	<0.0005
Tubular dilatation	0(0–1) A	0(0–1) A	3.5(2–4) B	1.5(1–2) C	51.044	<0.0005
Cast formation	0(0–0.75) A	0(0–1) A	4(2.25–4) B	1.5(1–2) C	54.227	<0.0005
Tubular vacuolation	0(0–1) A	0(0–1) A	4(2–4) B	1.5(1–2) C	46.877	<0.0005
Inflammatory cellular infiltrate	0(0–1) A	0(0–1) A	3(2–3.75) B	1(1–2) C	48.078	<0.0005
Tubulointerstitial fibrosis	0(0–1) A	0(0–1) A	3(2–4) B	1.5(1–2) C	52.222	<0.0005
Score	3 (2–3.75) A	3(2–4) A	19.5(17–21) B	9(7.25–10) C	67.305	<0.0005

Results are tabulated as median and interquartile range. Different letters = significant difference.

## Data Availability

All data is contained within the article.
